# Chinese herbal formula Tongxie Yaofang for diarrhea-predominant irritable bowel syndrome: study protocol for a randomized, multiple-blind, placebo-controlled trial

**DOI:** 10.1186/s13063-022-06142-x

**Published:** 2022-03-21

**Authors:** Shi-Bing Liang, Mei Han, Hong-Jie Cheng, Qiao-Yan Zhang, Nai-Wei Zhang, Bo-Yi Jia, Nicola Robinson, Jian-Ping Liu

**Affiliations:** 1 Centre for Evidence-Based Chinese Medicine, Beijing University of Chinese Medicine, Beijing, 100029 China; 2 Institute for Excellence in Evidence-Based Chinese Medicine, Beijing University of Chinese Medicine, Beijing, 100029 China; 3 Fangshan Hospital of Beijing University of Chinese Medicine, Beijing, 102400 China; 4 Institute for Health and Social Care, London South Bank University, London, SE1 0AA UK

**Keywords:** Tongxie Yaofang, Chinese herbal medicine, Irritable bowel syndrome, IBS-D, Randomized controlled trial

## Abstract

**Introduction:**

Diarrhea-predominant irritable bowel syndrome (IBS-D) is a bowel disease with a high incidence. It significantly reduces the quality of life of patients and affects the patient's daily activities and mental health. No specific therapeutic medications for IBS-D have been found. Published clinical trials suggest that Chinese herbal formula Tongxie Yaofang (TXYF) for IBS-D may be effective. However, high-quality clinical evidence supporting its use in IBS-D is still lacking. This trial aims to evaluate the therapeutic effects and safety of TXYF for IBS-D in adults.

**Methods/design:**

A randomized, multiple-blind, placebo-controlled trial will be conducted. It will consist of an 8-week intervention followed by a 3-month follow-up period.

The target sample size is 96 IBS-D patients aged 18 to 65 years. The eligible participants will be randomly allocated to either TXYF or placebo group in a ratio of 1:1. Participants in the experimental group will take TXYF granules, while participants in the control group will be given TXYF placebo granules. The primary outcome will be the degree of IBS symptom severity measured using the scale of IBS symptom severity score. The secondary outcomes include: (a) stool frequency, and (b) stool consistency measured using the Bristol stool scale, (c) quality of life measured using the scale of IBS-quality of life, (d) anxiety measured using the self-rating anxiety scale, (e) depression measured by the self-rating depression scale, and (f) the safety of using TXYF and placebo. Safety monitoring and assessment will be undertaken throughout treatment.

**Discussion:**

Chinese herbal formula TXYF consists of four Chinese herbs: *Atractylodes macrocephala Koidz.*, *Paeonia lactiflora Pall**.*, *Citrus × aurantium L**.*, and *Radix saposhnikoviae*. It has been used for diarrhea for hundreds of years and may have a potential benefit in treating adults with IBS-D, but due to lack of high-quality evidence, we designed a randomized, multiple-blind, placebo-controlled trial to evaluate its therapeutic effects and safety. This trial will provide important data to guide the clinical practice of TXYF for the treatment of IBS-D in adults.

**Trial registration:**

ISRCTN registry ISRCTN12453166. Registered on 23 March 2021.

## Introduction

Irritable bowel syndrome (IBS) is a functional gastrointestinal disorder, with a worldwide prevalence of approximately 11% [1]. The symptoms of IBS include chronic, recurrent, abdominal pain and discomfort, and altered bowel habits that occur in the absence of other organic gastrointestinal diseases [2]. It significantly reduces the quality of life of patients, their partners and caregivers, and affects daily activities and mental health [3, 4]. The underlying cause of IBS is thought to be multi-factorial, including: genetic factors, early life experience, psychological factors, prior gastroenteritis, food intolerance, and previous medication use [2, 5–8]. Based on the predominant stool patterns according to Rome VI criteria [9], IBS is classified into the following four types: constipation-predominant irritable bowel syndrome (IBS-C), diarrhea-predominant irritable bowel syndrome (IBS-D), mixed irritable bowel syndrome (IBS-M), and undefined irritable bowel syndrome. Of these, IBS-D is the most common type and accounts for up to 40% of patients with IBS [1, 10]. The specific pathogenesis of IBS has not been clarified clinically, and studies have shown that it may be related to brain and bowel dysfunction, gastrointestinal motility disorders, visceral hypersensitivity, psychosocial problems, and intestinal flora disorders [1, 11]. The treatment strategy of IBS-D includes antispasmodics for abdominal pain, antidiarrhoeals, nutritional intervention, and psychotherapy, but no specific therapeutic drugs that are able to treat all the symptoms have been found [12]. Some routine pharmacotherapies (e.g., antispasmodic drugs and antidiarrheal drugs) and alternative treatments (e.g., hypnotherapy and psychotherapy) [13] have been tried but have failed to achieve the desired clinical therapeutic effects.

IBS-D belongs to the category of diarrhea in traditional Chinese medicine (TCM) [14]. TCM may have potential in the treatment of IBS-D. Its therapies include oral traditional Chinese herbal formulas, external Chinese medicine treatments, and oral Chinese patent medicines. The most commonly used Chinese herbal formula is Tongxie Yaofang (TXYF) [15]. Chinese herbal formula TXYF was first recorded in the book named *Danxi Xinfa*, which is a comprehensive medical book edited in Yuan Dynasty (1271–1368) [16]. TXYF consists of four Chinese herbal medicines including *Bai Zhu (白术, Atractylodes macrocephala Koidz.)*, *Bai Shao (白芍,* *Paeonia lactiflora Pall**.)*, *Chen Pi (陈皮,* *Citrus × aurantium L**.)*, and *Fang Feng (防风, Radix saposhnikoviae)*. Clinically, TXYF usually has two dosage forms: granules and decoction. A series of studies have shown that TXYF can treat IBS-D by regulating the brain-gut axis, protecting the permeability of the intestinal mucosa, improving visceral hypersensitivity, and improving the body’s immune function [17–20]. Previous clinical trials [14, 21–24] have also demonstrated that TXYF is effective and safe for the treatment of IBS-D. However, to our knowledge, most trials have not been rigorously designed, especially in terms of the lack of randomization, blind and placebo control [14].

This randomized, multiple-blind, placebo-controlled trial is designed to evaluate the therapeutic effects and safety of TXYF in adults with IBS-D.

## Methods/design

### Study design

**Fig. 1:**
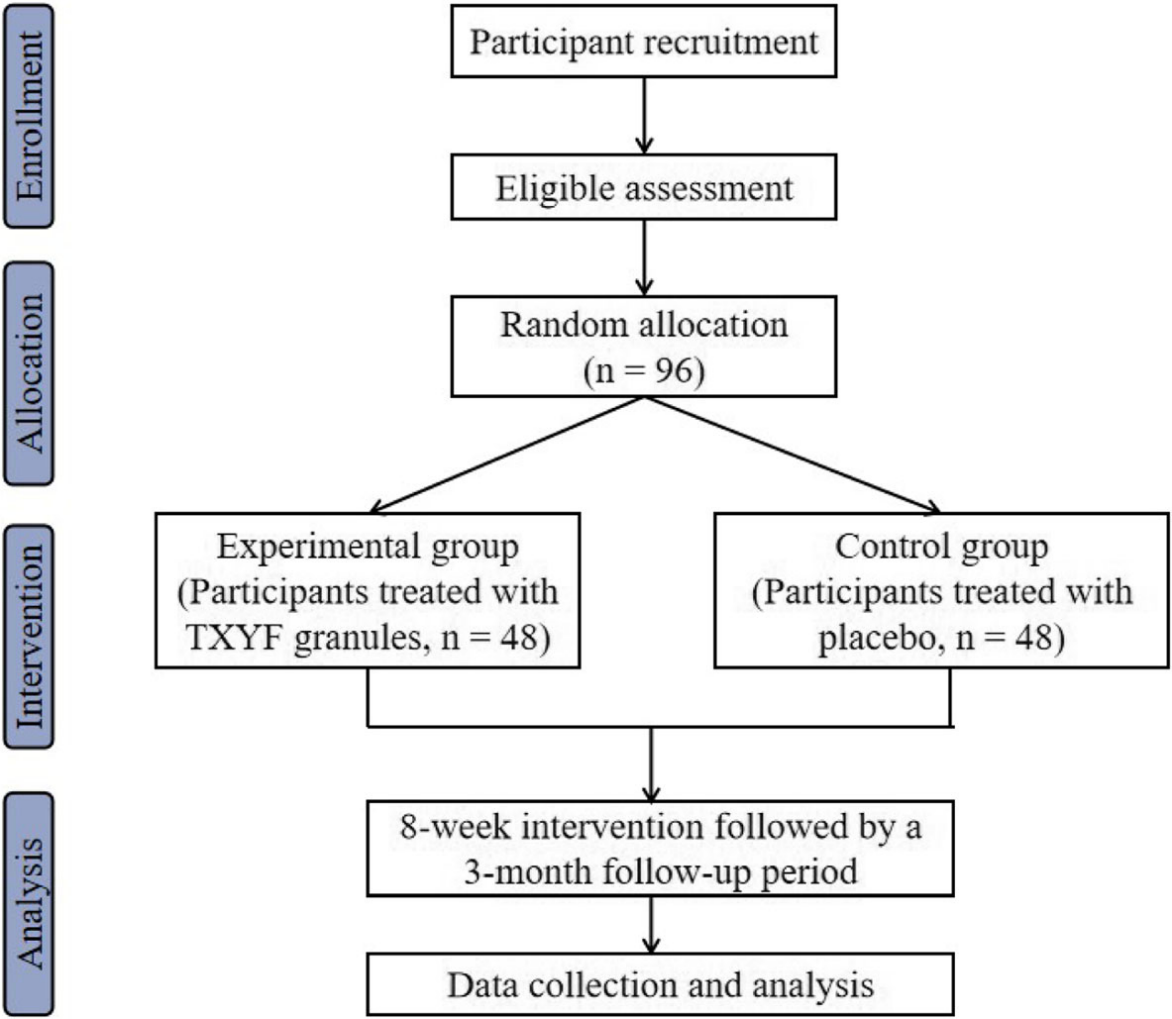
Study flow chart

This is a randomized, multiple-blind, placebo-controlled superiority trial, and the protocol for this trial has been prepared following the Standard Protocol Items: Recommendations for Interventional Trials (SPIRIT) guidelines (see Additional file 1) [25]. The eligible participants will be allocated randomly into the experimental or placebo control group in a ratio of 1:1. Both groups will have an 8-week intervention followed by a 3-month follow-up period. This trial protocol has been registered in the ISRCTN registry (Reference number: ISRCTN12453166) on the 23 March 2021. A flow diagram of the trial is shown in Fig. 1.

### Trial setting

We will recruit 96 IBS-D participants from outpatient or ward of a hospital in Beijing, China: Fangshan Hospital of Beijing University of Chinese Medicine (FSH-BUCM).

### Participants

#### Recruitment

Participants will be recruited through e-posters on social media (e.g., WeChat Moments) or posters in the hospital. The poster/e-poster information will include a brief description of the participants who would be eligible and details of activities and necessary contributions as participants in the trial. Participant expressing an interest to take part will have the opportunity to talk with the researchers who will introduce the trial protocol as well as the benefits and risks to each enrolled participant, and informed consent form will be mandatory before the patient is enrolled in the trial. The compliance of the participants will be improved by regular telephone follow-up.

#### Participant inclusion criteria

The included participants must meet all the following criteria: (a) patients aged between 18 and 65 years, male or female; (b) patients who meet IBS-D Rome IV diagnostic criteria [9]; (c) patients who meet Liver Depression and Spleen Deficiency Syndrome (Ganyu Pixu Zheng) from the diagnostic criteria for TCM syndromes (2017) [26]; (d) irritable bowel syndromes symptom severity score (IBS-SSS) ≥ 75 points; (e) patients who have not taken any medications related to the treatment of IBS-D at least 1 week before enrolling the trial and have not participated in other ongoing trials; (f) patients who have had a colonoscopy within 1 year and had an examination report; (g) patients who have accepted to participate in the trial voluntarily and have signed the informed consent form. The informed consent process complies with Good Clinical Practice (GCP); (h) long-term (at least 6 months) residence in the place where the treatment is given.

#### Participant exclusion criteria

Patients who meet any of the following items will be excluded: (a) IBS patients with alternating diarrhea and constipation (relying on patients recall); (b) patients with severe tumors or organic lesions in the heart, liver, or kidney; (c) patients with severe mental illness or language disorders that affect communication; (d) patients with severe tumors or organic lesions in gastrointestinal tract, such as pancreatitis, history of colon or rectal cancer, intestinal tuberculosis, ulcerative colitis or Crohn’s disease; (e) patients with metabolic diseases affecting gastrointestinal motility, such as hyperthyroidism; (f) those who with an allergic constitution or allergic to the composition of the studied medication; (g) patients with a history of gastrointestinal surgery; and (h) pregnant or lactating women and women planning to have a child or fertility treatment.

#### Criteria for participants who discontinue the trial

If a participant experiences aggravation of their disease, complications or serious adverse events, and the researcher judges that the participant is not suitable to continue the treatment, the patient should be allowed to discontinue. For participants who discontinue the trial, the researcher should record in detail the time and reason for discontinuing in the case report form (CRF).

### Intervention

#### The experimental group

**Fig. 2:**
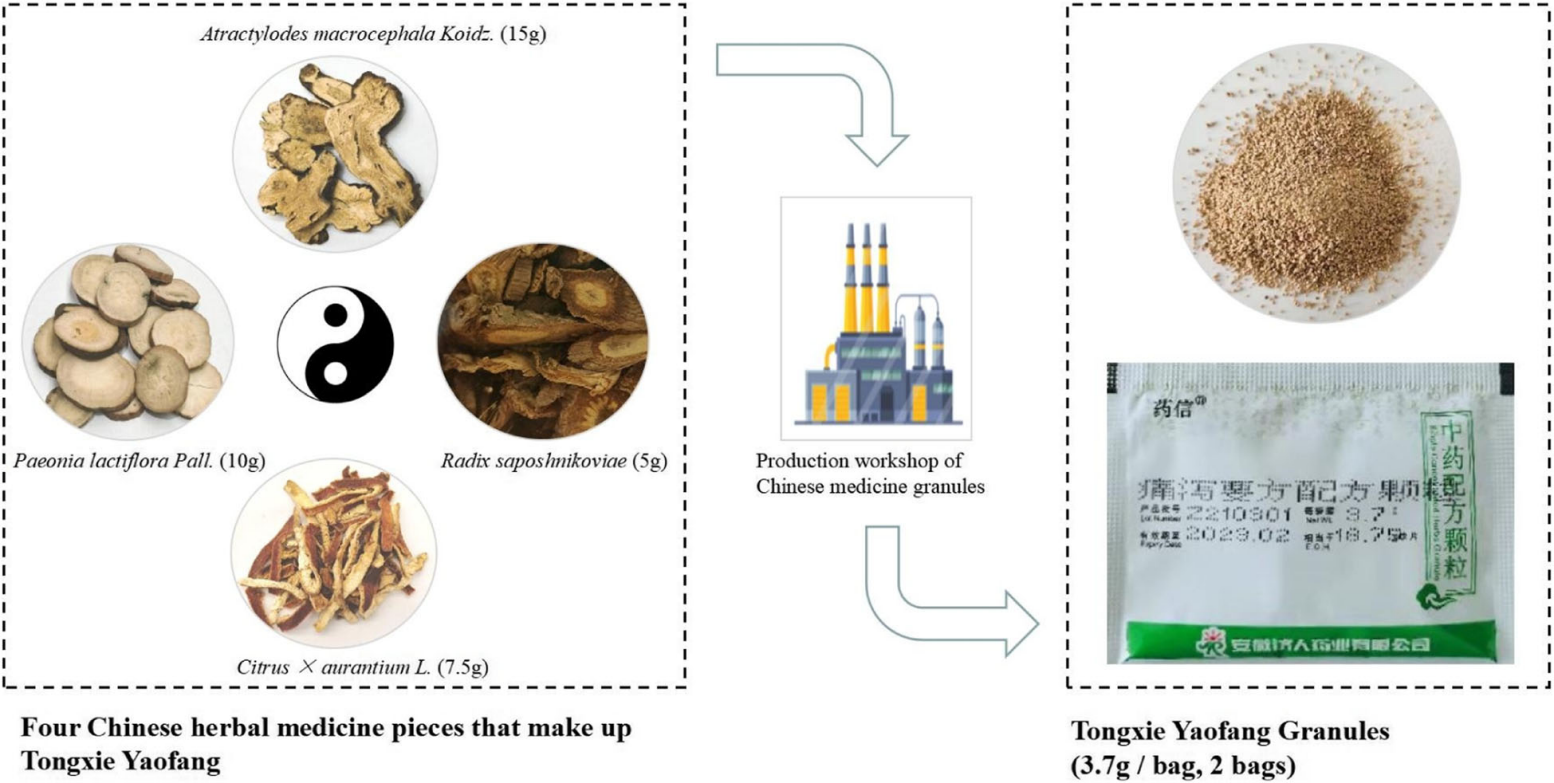
A diagrammatic sketch of the production process of Chinese medicine decoction pieces into granules

Participants will take TXYF granules manufactured by Anhui Jiren Pharmaceutical Co., Ltd. (Anhui, China). They will be instructed to take the granules orally before meals, one bag (3.7 g) twice daily, for 8 weeks. The granules are produced from four Chinese herbal pieces: *Bai Zhu (白术, Atractylodes macrocephala Koidz.)*, *Bai Shao (白芍,* *Paeonia lactiflora Pall**.)*, *Chen Pi (陈皮,* *Citrus × aurantium L**.)*, and *Fang Feng (防风, Radix saposhnikoviae)*. According to the records on the dosage of four Chinese herbal pieces from TXYF in *Danxi Xinfa* and the extraction rate of effective components in the process of granules production, we have determined the daily dosage of TXYF granules to be taken. A diagrammatic sketch of the production process of Chinese medicine decoction pieces into granules is presented in Fig. 2.

#### The control group

Participants will take TXYF placebo granules manufactured by Anhui Jiren Pharmaceutical Co., Ltd. (Anhui, China), take orally before meals, one bag (3.7 g) twice daily, for 8 weeks. The placebo is made of lactose, flour, sucrose, edible caramel pigment, bitter and aspartame, etc. Its appearance, taste, and specification are consistent with TXYF granules to the greatest extent possible. In terms of outer packing, the placebo and TXYF granules will be exactly the same.

#### Concomitant treatment

If the participant is unable to tolerate the IBS-D symptoms or is dissatisfied with the therapeutic effects of TXYF during the treatment, pinaverium bromide (the first-line recommended drug in American Gastroenterological Association Institute Guidelines on the Pharmacological Management of Irritable Bowel Syndrome [27]) or other medications recommended by guidelines will be allowed to be taken by the patient. No matter what medications the patient uses, the medication’s name, dosage and duration of administration will be recorded in detail in CRF.

#### Experimental medication preparation and quality control

Anhui Jiren Pharmaceutical Co., Ltd. (Anhui, China) is responsible for the production and quality control of TXYF granules and placebo.

### Randomization, allocation concealment, and blinding

A sequence of random numbers was generated by the R version 4.0.3 software and performed by the Centre for Evidence-based Chinese Medicine, Beijing University of Chinese Medicine (CEBCM-BUCM). The eligible participants will be randomly assigned to either the experimental (TXYF) or control (placebo) group in a ratio of 1:1. Allocation concealment will be done, using the method of “coding the drug packaging,” to minimize selection bias [28–30]. The sequence of randomization and allocation will be kept blind at all times to all participants and clinician until the trial has been completed. The placebo appearance, taste, and specification will be similar to TXYF granules as far as possible. The placebo and TXYF granules will have exactly the same outer packaging. Only the designated persons (SB Liang and JP Liu from the CEBCM-BUCM) will have access to the sequence of randomization and allocation during the trial, and they will not share the relevant information until such a time where the study has concluded and all of the data has been collected (unless serious adverse event occurs).

During the trial, if a participant experiences a serious adverse event or needs emergency rescue, the clinician can request the clinical research associate or the main researcher to disclose the intervention the participant has received. Once it is decided to disclose the intervention the participant has received, the participant will be recorded as a withdrawal case. Once the number of participants whose intervention is disclosed reaches or exceeds 20% of the required sample size, the trial can be considered to be a failure in blinding the participants and clinicians [31].

Furthermore, the outcome assessors collecting the outcome data were blinded to the allocation of the interventions too, throughout the study. Before completing the statistical analysis, the statistical analyst is also unable to know whether the participant is taking TXYF or placebo.

### Outcomes

We referred to the 2017 Consensus on Diagnosis and Treatment of Irritable Bowel Syndrome in TCM [26], outcomes in the relevant registered or published clinical trials, as well as the actual clinical diagnosis and treatment of IBS-D, to determine the outcomes to be evaluated in our this trial.

#### Primary outcomes

The degree of IBS symptom severity, measured using the scale of irritable bowel syndromes symptom severity score (IBS-SSS) [32]. The disease severity will be divided into four levels base on IBS-SSS: normal (the score < 75), mild (75 ≤ the score < 175), moderate (175 ≤ the score < 300), and severe (the score ≥ 300). If the disease severity remains at the original (baseline) level or changes to higher level (e.g., from mild to moderate) after treatment, it will be judged as no response; otherwise, it will be considered response. The response rate = (total number of participants − number of no response participants)/total number of participants × 100%. Finally, we will not only compare the scores after treatment between groups, but also compare the response rate.

#### Secondary outcomes

(a) Stool frequency: the average daily number of spontaneous defecation recorded in the week before each time-point; (b) stool consistency measured using the Bristol stool scale [33]; (c) quality of life measured using the scale of IBS-quality of life (IBS-QOL) [34]; (d) anxiety measured using the self-rating anxiety scale (SAS) [35]; (e) depression measured using the self-rating depression scale (SDS) [36]; (f) the safety using TXYF and placebo, which will be evaluated in the following aspects: (1) routine examination (blood routine, urine routine, stool routine + occult blood (OB)); (2) biochemical indexes including liver function (aspartate aminotransferase (AST) and alanine aminotransferase (ALT)), kidney function (blood urea nitrogen (BUN), creatinine); (3) heart electrical activity measured using electrocardiogram; (4) adverse events, such as rash, constipation, or other special symptoms, recorded at any time during the treatment by the patient: incidence of adverse events = (number of adverse events/total cases) × 100%; (5) severe adverse events, such as loss of function or disability, life-threatening or even death, recorded at any time during the treatment by the researchers: incidence of severe adverse events = (number of severe adverse events/total cases) × 100%.

#### Patient timelines for participants

The schedule of enrolment, treatment and assessments is presented in Table 1.

**Table 1:**
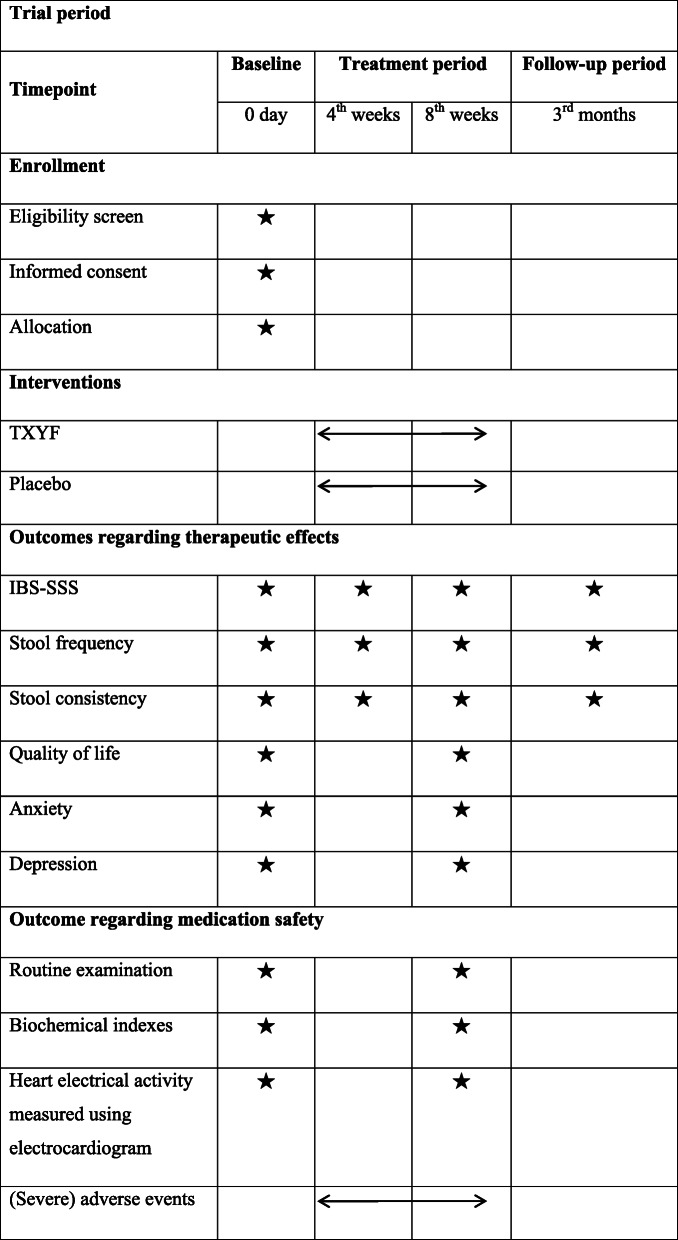
Schedule of enrollment, treatment and outcome assessment

*TXYF* Tongxie Yaofang Granules, *IBS-SSS* irritable bowel syndromes symptom severity score

Routine examination including blood routine, urine routine, stool routine, and occult blood test (OBT); biochemical indexes including liver function (aspartate aminotransferase (AST) and alanine aminotransferase (ALT)) and kidney function (blood urea nitrogen (BUN), creatinine)

### Sample size

According to previous studies [37], it is estimated that TXYF can produce efficacy response (according to IBS-SSS) in 73% of participants with eight weeks treatment. Under the same treatment duration and evaluation criteria used in this trial, no results of placebo treatment of IBS-D were found. However, based on previously published relevant studies [38, 39], we expect TXYF to have a 30% higher response rate than placebo in the primary outcome, and superiority margin is expected to be 10% (*δ* = 10%). The ratio of the TXYF group to the control group was 1:1. So, the total sample size of 96 is based on the following formula [40], when *α* = 0.05, power = 0.8, and the estimated loss of participants is 10% [31]. That is, the number of participants to be enrolled in the trial should be 48 in each group.

$$ n={\left({Z}_{1\hbox{-} \alpha / 2}+{Z}_{1\hbox{-} \beta}\right)}^2\left[{P}_1\left( 1\hbox{-} {P}_1\right)+{P}_2\left( 1\hbox{-} {P}_2\right)\right]/{\left(P 1\hbox{-} P 2\hbox{-} \delta \right)}^2 $$

Where *n* denotes the sample size of each group, the value of *Z*_*1-α*_ and *Z*_*1-β*_ needs to consult the Z value table, *P*_*1*_ denotes the rate of the experimental group and *P*_*2*_ represents the rate of the control group, and *δ* represents the superiority margin. Therefore, when calculating the sample size of this trial, the value of *Z*_*1-α*_ is 1.96, the value of *Z*_*1-β*_ is 0.842, the value of *P*_*1*_ is 73%, the value of *P*_*2*_ is 43%, and the value of ***δ*** is 10%.

### Data collection and management

Data collection will be performed during the treatment period, as well as the follow-up period. The data will be recorded in the CRF and entered into an electronic data management software (e.g., EpiData Software). All private and sensitive data (e.g., identification information from participants) will be coded.

### The trial quality control

The regulations for trial quality control are as follows: (a) the designated personnel from FSH-BUCM are responsible for the monitoring of the entire trial and (b) the designated personnel from CEBCM-BUCM assisted in monitoring the entire trial.

The content of quality control includes the following: (a) ensure that the trial process complies with the protocol and ensure the truth, accuracy, and completeness of the data and (b) monitor the progress of the trial, ensure the timely recording and reporting of adverse events, and ensure the informed consent and protection of the participants.

### Statistical analysis

Data analysis will follow the trial statistical analysis plan and be performed by the designated personnel from CEBCM-BUCM. All data will be processed by statistical analyses with SPSS or other appropriate software.

#### Principle of analysis

All analyses will be by the intention-to-treat (ITT) principle which included all randomized patients who received the TXYF or its placebo and returned for at least one follow-up appointment. The per-protocol set (PPS) analysis for the primary outcomes is being conducted in conjunction with the ITT analysis. When the results of ITT and PPS analysis are conflict, the results of ITT analysis will be given preference. When the results of ITT and PPS analysis are consistent, the reliability of the results can be increased. ITT will be used for the analysis of baseline characteristics, and safety set (SS) will be used for the safety evaluation.

#### Statistical description and statistical analysis methods

With regard to continuous outcomes, Q-Q plots will be used to evaluate normality assumptions. Continuous outcomes conforming to the normal distribution will be presented as mean ± standard deviation (SD) and non-conforming to the normal distribution will be presented as median and interquartile range (IQR) [41]. For both primary continuous outcomes and secondary continuous outcomes, simple or multiple linear regression will be applied to estimate the difference between TXYF group and placebo group. Two preadjusted analyses will be conducted, if appropriate. The first preadjusted regression analysis included only baseline variables (e.g., age, gender, smoking and drinking). The second preadjusted analysis will also adjust for concomitant treatment, a variable thought to be associated with outcome but not affected by treatment allocation.

Categorical data will be expressed by the number and percentage of occurrences. For primary and secondary categorical outcomes, differences will be compared using chi-squared tests and, if appropriate and necessary, logistic regression. The preadjusted analyses will be conducted that adjust for the same covariates as those described for the continuous outcomes.

Estimates of treatment effect will be reported with 95% confidence intervals. Two-tailed *P*-values ≤ 0.05 will be considered statistically significant.

### Ethics

This trial will be completed in accordance with the ethical principles in the Declaration of Helsinki and the Good Clinical Practice (GCP) Guideline. It has been approved by Medical Ethics Committee from FSH-BUCM approved on 4 February 2021 (ref: FZY LK-2021-002). CEBCM-BUCM will monitor and audit this trial.

If the trial’s protocol needs to be amended, the revisions will be discussed, communicated and concluded by the main investigators. The revised protocol will also be submitted to the Medical Ethics Committee for approval, and an application for revisions will also be submitted to the trial registry.

## Discussion

Irritable bowel syndrome is the most common functional gastrointestinal disorder characterized by abdominal pain/discomfort which is relieved by defecation and associated with altered stool frequency and/or consistency, in the absence of structural, microbiological, and biochemical abnormalities [2, 7]. The occurrence of this disease is not associated with structural or biochemical abnormalities that are detectable with the current routine diagnostic tools [42]. IBS-D with a high population prevalence that accounts for about 40% of all IBS patients. IBS-D significantly reduces the quality of life of patients and affects the patient’s daily activities and mental health. The specific pathogenesis of IBS-D has not been clarified clinically, and no specific therapeutic medications that can treat all the symptoms of IBS-D have been found.

IBS-D belongs to the category of diarrhea in TCM [14]. According to the diagnostic criteria for TCM syndromes [26], IBS-D can be divided into multiple syndrome types, such as Liver Depression and Spleen Deficiency Syndrome, Spleen Deficiency and Dampness Excess Syndrome (Pixu Shisheng Zheng), and Spleen-yang and Kidney-yang Deficiency Syndrome (Pishen Yangxu Zheng). Of these, Liver Depression and Spleen Deficiency Syndrome account for the highest proportion (85.6%) of patients with IBS-D [43]. Some herbal medicines may improve the symptoms of irritable bowel syndrome [44]. TXYF is one of the most commonly used Chinese herbal medicines for the treatment of IBS-D [16]. TXYF was first described by a book named *Danxi Xinfa* from Yuan Dynasty (1271-1368) and consists of *Atractylodes macrocephala Koidz.*, *Paeonia lactiflora Pall**.*, *Citrus × aurantium L**.*, and *Radix saposhnikoviae*. An animal experiment demonstrated that TXYF could increase the pain threshold and decrease the number of fecal pellets in IBS rats [45]. Also, TXYF has been shown in a clinical trial to demonstrate the therapeutic effects of anti-pain and anti-diarrhea on IBS-D patients [46].

Although some relevant studies have been carried out to verify the therapeutic effects and safety of TXYF in the treatment of IBS-D, to the best of our knowledge, the high-quality clinical trial is still lacking and not enough. The trial will allow in-depth analyses of mediating and moderating effects for different outcomes of TXYF in the treatment of IBS-D. Evidence regarding the therapeutic effects and safety of TXYF will be provided, and if effective and safe, this has the potential to be implemented in routine care to relieve the symptoms of IBS-D and improve the overall quality of life of patients. Success of this trial will present the justification and impetus for conducting a large-scale clinical trial to further consolidate the evidence for the use of TXYF in the treatment of IBS-D.

In conclusion, the results of this trial are expected to provide a high-quality and reliable evidence for the therapeutic effects and safety of the Chinese herbal formula TXYF for the treatment of IBS-D patients who also meet the diagnostic criteria of Liver Depression and Spleen Deficiency Syndrome.

## Trial status

The protocol version is V6.0/202011. The participants’ recruitment started in April 2021 and is planned to continue until September 2022.

## Data Availability

The datasets supporting the findings of our study are available from the corresponding author upon request.

## Abbreviations

TXYF: Tongxie Yaofang
IBS-C: Constipation-predominant irritable bowel syndrome
IBS-D: Diarrhea-predominant irritable bowel syndrome
IBS-M: Mixed irritable bowel syndrome
TCM: Traditional Chinese medicine
SPIRIT: The Standard Protocol Items: Recommendations for Interventional Trials
IBS-SSS: Irritable bowel syndromes symptom severity score
GCP: Good Clinical Practice
CRF: Case report form
ITT: Intention-to-treat
CEBCM-BUCM: Centre for Evidence-based Chinese Medicine, Beijing University of Chinese Medicine
IBS-QOL: IBS-quality of life
SDS: The self-rating depression scale
OB: Occult blood
AST: Aspartate aminotransferase
ALT: Alanine aminotransferase
BUN: Blood urea nitrogen
FSH-BUCM: Fangshan Hospital of Beijing University of Chinese Medicine
LOCF: Last-observation-carried-forward
PPS: The per-protocol set
SS: Safety set
